# Outcomes after elective total aortic arch replacement: Does obesity matter?

**DOI:** 10.1016/j.xjon.2026.101736

**Published:** 2026-03-20

**Authors:** Vicente Orozco-Sevilla, Michael Tyler Guinn, Ivan Murrieta-Alvarez, Veronica A. Glover, Susan Y. Green, Subhasis Chatterjee, Scott A. LeMaire, Marc R. Moon, Joseph S. Coselli

**Affiliations:** a Division of Cardiothoracic Surgery, Michael E. DeBakey Department of Surgery, Baylor College of Medicine, Houston, Tex; b The Texas Heart Institute at Baylor College of Medicine, Houston, Tex; c Department of Cardiovascular Surgery, CHI St Luke's Health—Baylor St Luke's Medical Center, Houston, Tex; d Bioengineering Department, Rice University, Houston, Tex; e Office of Surgical Research, Michael E. DeBakey Department of Surgery, Baylor College of Medicine, Houston, Tex; f Departments of Investigational Medicine and Cardiothoracic Surgery, Geisinger College of Health Sciences, Danville, Pa; g Cardiovascular Research Institute, Baylor College of Medicine, Houston, Tex

**Keywords:** aortic arch, obesity, postoperative complications, body mass index, cardiovascular surgical procedures

## Abstract

**Objective:**

Obesity is a chronic disease linked to high mortality and morbidity after cardiac procedures, but its relationship to outcomes after total aortic arch replacement (TAR) remains unclear. We examined TAR data to determine whether obesity is associated with greater perioperative risk.

**Methods:**

Among 787 TARs performed from 1990 to 2023, we excluded patients who lacked body mass index (BMI) data, had nonelective or nonstandard repairs, or were underweight (BMI <18.5 kg/m^2^). The remaining 521 patients did not have obesity (18.5 ≤BMI <30; n = 389) or did (BMI ≥30; n = 132). Patients with obesity were class 1 (30 ≤ BMI < 35), class 2 (35 ≤ BMI < 40), or class 3 (BMI ≥40). We compared preoperative and perioperative variables for BMI ≥30 (obesity) versus BMI <30. Adverse events were operative mortality, persistent stroke, spinal cord deficit, and renal failure. We performed multivariable logistic regression and Kaplan-Meier survival analysis.

**Results:**

Patients with obesity had greater rates of obstructive sleep apnea, transient spinal cord deficit, and acute renal dysfunction than patients with normal weight. During repair, the use of antegrade cerebral perfusion often exceeded 30 minutes in patients with obesity. Operative mortality and adverse events did not differ among BMI or obesity groups. Greater BMI was not predictive of operative mortality, whereas chronic kidney disease (odds ratio, 2.28; *P* = .01), pulmonary disease (1.87, *P* < .001), longer aortic clamp time (1.01, *P* = .03), and antegrade cerebral perfusion time >30 minutes (2.17, *P* = .047) were. Survival did not differ significantly by obesity status.

**Conclusions:**

Obesity was not associated with operative mortality or adverse events in patients who underwent TAR. Therefore, patients should not be deemed ineligible for TAR on the basis of obesity alone.

**Figure:**
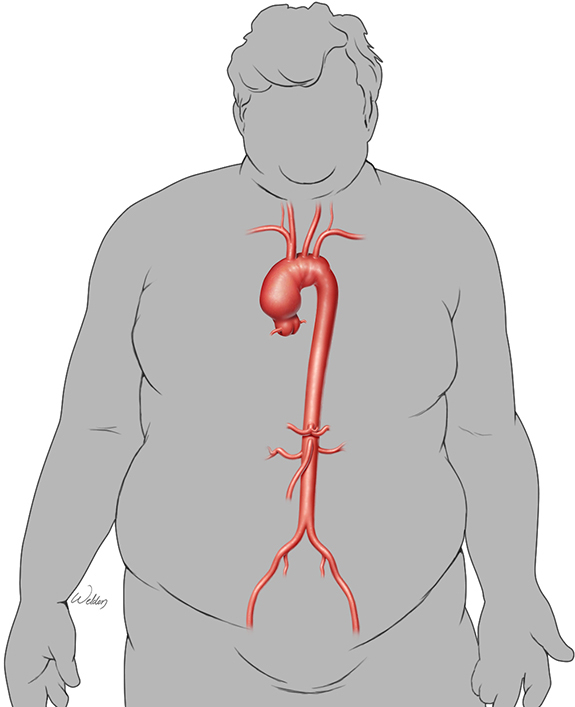
In patients undergoing total arch replacement, obesity does not increase operative risk.

**Central Message**

Obesity is associated with greater risk in patients undergoing cardiac procedures. However, we did not associate obesity with greater operative mortality or morbidity after total arch replacement.

**Perspective**

Obesity is correlated with greater morbidity and mortality after cardiac surgery. However, its effect on outcomes in total arch replacement (TAR) is unclear. In a multivariate analysis, we found no association between obesity and post-TAR operative mortality or morbidity, including persistent stroke, spinal cord deficit, and renal failure necessitating dialysis.

Obesity is a chronic health condition that continues to grow, affecting nearly 10% of the world's population.^1^^,^^2^ The implications of obesity for health are widespread because it contributes to diabetes mellitus,^3^ hypertension,^4^ cancer,^5^^,^^6^ and cardiovascular diseases like ischemic heart disease.^7^ In patients who undergo surgery, obesity is correlated with greater rates of mortality and major morbidity (eg, renal failure) after cardiac procedures.^8^ However, the effects of obesity on aortic arch replacement outcomes are not fully elucidated. We analyzed body mass index (BMI) data from patients who underwent elective total aortic arch replacement (TAR) to determine whether obesity is associated with greater risk of perioperative mortality and morbidity.

## Methods

### Study Protocol and Patient Cohort

Baylor College of Medicine's institutional review board approved our clinical research protocol (#H18095) on February 21, 2006. For patients who underwent TAR after protocol approval, clinical data were collected prospectively, and informed consent was obtained whenever possible. A waiver of consent was approved for patients who underwent surgery before the protocol was approved or whose illness prevented them from providing consent and who had no family members available to provide consent for them. Data collection before 2006 was retrospective.

Of the 3238 open aortic arch repairs performed by our single practice from 1990 to 2023, 787 (24.3%) were TARs. We excluded patients who had no BMI data (n = 34), whose TAR was nonelective (n = 205) or did not involve median sternotomy (n = 8), or who were underweight according to the World Health Organization's BMI classification (BMI <18.5 kg/m^2^; n = 19).^9^ The remaining 521 patients were categorized as healthy weight (18.5 ≤ BMI < 25; n = 165), overweight (25 ≤ BMI < 30; n = 224), or obese (BMI ≥30; n = 132); patients with obesity were categorized as class 1 (30 ≤ BMI < 35; n = 94), class 2 (35 ≤ BMI < 40; n = 29), or class 3 (BMI ≥40; n = 9). We compared patients with obesity (BMI ≥30; n = 132) and without obesity (18.5 ≤ BMI <30; n = 389).

### Study Definitions and Follow-up

All data were collected by using standard definitions.^10^^,^^11^ Heritable thoracic aortic disease was defined as a documented connective tissue disorder (eg, Marfan, Loeys-Dietz) with or without formal clinical evaluation (ie, Ghent criteria) or genetic testing, or undergoing thoracic aortic repair at age ≤50 years. Pulmonary disease, a composite variable, comprised asthma, chronic obstructive pulmonary disease, obstructive sleep apnea, and any history of lung transplant, spontaneous pneumothorax, or tuberculosis. Each TAR incorporated at least 2 brachiocephalic arteries into the repair. We simplified the description of brachiocephalic artery reattachment during TAR to include 2 major types: an “island” approach, in which native arteries are reimplanted as a patch, and a “graft” approach, in which a bypass graft (branch graft) replaces a portion of these arteries. Reattachments involving both island and graft techniques were categorized by the dominant approach (ie, 2 of 3 vessels). Proximalization of repair described a distal anastomosis performed at the anatomic level between the left common carotid and left subclavian arteries. Following societal guidelines, operative death comprises 30-day death, in-hospital death, and death after transfer to an acute care facility.^12^ Adverse event was a composite end point comprising operative death, neurologic deficit, and renal failure necessitating dialysis at discharge.

Survivors of the operations were contacted for clinical follow-up approximately 60 days after repair and yearly thereafter. Postoperative surveillance information was obtained through clinic visits, telephone interviews, or written correspondence. The Social Security Death Index (up to 2011) and internet obituary searches were used to identify deaths among patients who were lost to follow-up. Patients’ overall survival was defined as the duration from the date of surgery to the date of the most recent survival status update.

### Surgical Techniques

**Figure 1:**
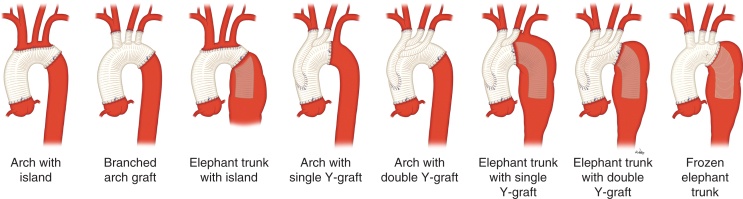
Basic graft configurations used in total aortic arch repair: a tube graft with an island or patch reattachment, anatomic branched grafts, single and double Y-grafts, and options for classic and frozen elephant trunks. Open repair facilitates a patient-specific approach. The graft configurations shown are not an exhaustive representation. Used with permission of Baylor College of Medicine.

We have previously investigated various surgical techniques for total arch replacement, which have evolved over time (Figure 1, Table E1).^11^^,^^13^, ^14^, ^15^ All repairs involved median sternotomy and hypothermic circulatory arrest (HCA). For standard repairs in patients without obesity, arterial cannulation for cardiopulmonary bypass was performed at the right axillary, innominate, or femoral artery, or by directly cannulating the ascending aorta or another arterial site. In contemporary repair (≥2006), HCA at a moderate target temperature (24-26 °C, nasopharyngeal), is commonly used with antegrade cerebral perfusion (ACP) after either the right axillary artery or the innominate artery is cannulated. Bilateral ACP is preferred to unilateral ACP. Near-infrared spectroscopy is used to assess cerebral oxygenation in real time. Repair often extended beyond the left subclavian artery to include an elephant trunk or frozen elephant trunk approach. The introduction of collared grafts in recent years has shifted the distal anastomosis to a more proximal location (eg, between the left common carotid and left subclavian arteries instead of the traditional location distal to the left subclavian artery) and allowed greater flexibility in the management of the left subclavian artery.^13^ As needed, repair addresses the aortic root or aortic valve.

For patients with obesity, it is sometimes more difficult to obtain high-quality imaging studies because of logistical limitations (eg, size of patient within imaging machine) and signal attenuation related to fat mass.^16^ Transesophageal echocardiography is an imaging option in acute cases. In patients with obstructive sleep apnea, we ensure that they adhere to continuous positive airway pressure therapy and instruct them to bring their devices to the hospital for postoperative use. Compared with our standard approach, a larger table, longer surgical instruments, modified patient positioning, and enhanced lighting are typically needed during repair.^17^ Larger arterial (eg, 22-F) and venous drainage cannulas provide more blood flow during cardiopulmonary bypass, along with greater pump flow rates (6.0-7.0 L/min). Ensuring adequate blood oxygenation sometimes necessitates adding a second oxygenator to the pump to provide more surface area. This approach aims to overcome high oxygen consumption and increased carbon dioxide production. Monitoring temperature in remote locations (eg, the bladder) may be less accurate because a larger body takes longer to cool. Cannulation can be complicated by a fat pad near the right axillary artery and sometimes must be shifted to the innominate artery or another central artery. Closing the sternum in a patient with obesity can be more difficult, and the sequence of closing becomes more important. Retention sutures (ie, mattress sutures), double-wire closure, or the use of titanium sternal plates may be needed for effective sternal closure. A support bra, to take tension off the midline incision, and a wound vacuum may aid healing. We maintain a cautious approach to extubation in patients with known obstructive sleep apnea, balancing the benefits of early extubation (eg, earlier mobilization) against the risk of reintubation. We reinitiate continuous positive airway pressure therapy when clinically safe.

### Statistical Analysis

Statistical analyses were performed with IBM SPSS Statistics 28 (IBM Corp), SAS version 9.4 (SAS Institute, Inc), and R, version 4.2.2, from The R Project for Statistical Computing. Continuous variables are presented as median [quartile 1-quartile 3]. Categorical variables are presented as numbers and percentages. Univariate comparisons were conducted with the Pearson χ^2^ test, Fisher exact test, or Wilcoxon rank-sum test.

Multivariable logistic regression models were developed from preoperative and select operative variables with clinical relevance and univariate associations with major morbidity or mortality at *P* ≤ .1. We entered these variables into the model, then used a backward selection method with a removal *P* value of .15 to build the final model. In addition, BMI was modeled as a continuous exposure using a restricted cubic spline with 4 degrees of freedom to identify linear and nonlinear associations with operative death. Predicted probabilities of operative death were obtained from the multivariable logistic regression whereas other preoperative covariates were held at their baseline values. We graphed the adjusted association between BMI and operative death as a marginal-effects plot across the observed BMI range, centered at BMI = 25 (reference), to illustrate increases or decreases in the predicted probability relative to that value. The Kaplan-Meier method estimated postoperative survival rates, which were compared between groups by using the log-rank test.

## Results

### Preoperative Characteristics

Compared with the patients without obesity (BMI <30; n = 389), patients with obesity (BMI ≥30; n = 132) were more likely to be male (70.5% vs 59.9%; *P* = .03), more often had obstructive sleep apnea (28% vs 7.5%, *P* < .001), and trended toward more frequent hyperlipidemia (50.0% vs 40.6%, *P* = .06) (Table 1).

**Table 1:** Preoperative characteristics stratified by obesity

Variable	All (N = 521)	BMI <30 (n = 389)	BMI ≥30 (n = 132)	*P*
Age, y	65 [56-72]	66 [56-72]	65 [56-71]	.2
Male	326 (62.6)	233 (59.9)	93 (70.5)	.03
Heritable thoracic aortic disease	108 (20.7)	84 (21.6)	24 (18.2)	.4
Marfan syndrome	57 (10.9)	47 (12.1)	10 (7.6)	.2
Aortic aneurysm without dissection	283 (54.3)	217 (55.8)	66 (50.0)	.2
Chronic proximal aortic dissection	238 (45.7)	172 (44.2)	66 (50.0)	.2
DeBakey type I	225 (43.2)	162 (41.6)	63 (47.7)	.2
DeBakey type II	11 (2.1)	9 (2.3)	2 (1.5)	.6
Localized dissection	3 (0.6)	1 (0.3)	2 (1.5)	.1
Proximal (root, ascending, or arch) aortic diameter, max, cm	5.8 [5.1-6.6]	5.8 [5.1-6.6]	5.8 [5.1-6.5]	.7
	(n = 478)	(n = 354)	(n = 124)	
Distal (descending thoracic or thoracoabdominal) aortic diameter, max, cm	5.5 [4.6-6.5]	5.5 [4.6-6.5]	5.6 [4.7-6.5]	.8
	(n = 390)	(n = 286)	(n = 104)	
Hypertension	450 (87.4)	330 (85.9)	120 (91.6)	.1
Hyperlipidemia	223 (43.0)	157 (40.6)	66 (50.0)	.06
Diabetes	42 (8.1)	29 (7.5)	13 (9.8)	.4
Coronary artery disease	187 (35.9)	139 (35.7)	48 (36.4)	.9
Cerebrovascular disease	72 (13.8)	57 (14.7)	15 (11.4)	.3
Chronic kidney disease	139 (26.7)	103 (26.5)	36 (27.3)	.9
	(n = 486)	(n = 359)	(n = 127)	
BMI	27 [24-30]	26 [23-28]	33 [31-35]	<.001
Pulmonary disease	200 (38.4)	136 (35.0)	64 (48.5)	.006
COPD	109 (20.9)	81 (20.8)	28 (21.2)	.9
Obstructive sleep apnea	66 (12.7)	29 (7.5)	37 (28.0)	<.001
Current or former tobacco use	340 (65.3)	254 (65.3)	86 (65.2)	>.99
Symptoms of aneurysm				
Acute (any)	10 (1.9)	5 (1.3)	5 (3.8)	.1
Chronic (any)	295 (56.6)	215 (55.3)	80 (60.6)	.3
Prior open proximal aortic repair	251 (48.2)	184 (47.3)	67 (50.8)	.5

Values are n (%) or median [quartile 1-quartile 3]. *BMI*, Body mass index; *COPD*, chronic obstructive pulmonary disease.

### Operative Details

Most repairs in patients with obesity were performed in 2006 and later (Table 2, Table E1). There were few differences between patients with and without obesity, although patients with obesity had longer HCA duration (59 vs 54 minutes, *P* = .03) and more commonly had bilateral ACP (71.2% vs 53.0%, *P* < .001) and ACP exceeding 30 minutes (78.8% vs 63.2%, *P* < .001).

**Table 2:** Operative details of elective repair, stratified by obesity

Variable	All (N = 521)	BMI <30 (n = 389)	BMI ≥30 (n = 132)	*P*
Reoperation	270 (51.8)	198 (50.9)	72 (54.5)	.5
Surgical era				
<2006	153 (29.4)	129 (33.2)	24 (18.2)	.001
≥2006	368 (70.6)	260 (66.8)	108 (81.8)	.001
Arterial cannulation site (initial)				
Direct aorta (ascending/arch)	86 (16.5)	69 (17.7)	17 (12.9)	.2
Right axillary	229 (44.0)	166 (42.7)	63 (47.7)	.3
Innominate	112 (21.5)	81 (20.8)	31 (23.5)	.5
Right common carotid	20 (3.8)	12 (3.1)	8 (6.1)	.1
Femoral	68 (13.1)	56 (14.4)	12 (9.1)	.1
Other/unknown	6 (1.2)	6 (1.5)	0	.2
Primary brachiocephalic artery reattachment approach				
Island (≥2 vessels)	277 (53.2)	208 (53.5)	69 (52.3)	.8
Graft (≥2 vessels)	229 (44.0)	172 (44.2)	57 (43.2)	.8
LSCA management				
Preoperative bypass/transposition	31 (6.0)	20 (5.1)	11 (8.3)	.3
Intraoperative bypass/transposition	136 (26.1)	104 (26.7)	32 (24.2)	.6
Reimplantation or island	201 (38.6)	152 (39.1)	49 (37.1)	.7
Native (not incorporated)	146 (28.0)	108 (27.8)	38 (28.8)	.8
Other	7 (1.3)	5 (1.3)	2 (1.5)	.8
Aortic repair details (proximal)				
Isolated AV replacement	85 (16.3)	55 (14.1)	30 (22.7)	.02
Aortic root replacement	62 (11.9)	46 (11.8)	16 (12.1)	.9
Aortic repair details (distal)				
Proximalization of repair∗	190 (36.5)	134 (34.4)	56 (42.4)	.1
Reverse ET	20 (3.8)	11 (2.8)	9 (6.8)	.04
ET	393 (75.4)	295 (75.8)	98 (74.2)	.7
Classic	266 (51.1)	202 (51.9)	64 (48.5)	.5
Frozen	127 (24.4)	93 (23.9)	34 (25.8)	.7
Perfusion and ischemic times, min				
Aortic clamp time	54 [27-80]	54 [27-82]	53 [31-76]	>.9
	(n = 434)	(n = 322)	(n = 112)	
CPB time	138 [107-182]	136 [104-180]	143 [117-191]	.2
HCA systemic time	55 [44-69]	54 [43-67]	59 [46-73]	.03
Lowest nasopharyngeal temperature, °C	21 [15-23]	20 [14-23]	22 [17-24]	.005
Cerebral perfusion				
None	35 (6.7)	30 (7.7)	5 (3.8)	.1
RCP (any)	105 (20.2)	88 (22.6)	17 (12.9)	.02
ACP (any)	400 (76.8)	287 (73.8)	113 (85.6)	.005
ACP and RCP	19 (3.6)	16 (4.1)	3 (2.3)	.3
Bilateral ACP	300 (57.6)	206 (53.0)	94 (71.2)	<.001
ACP time, min	58 [44-73]	56 [43-72]	60 [47-74]	.1
	(n = 400)	(n = 287)	(n = 113)	
ACP time >30 min	361 (67.1)	251 (63.2)	104 (78.8)	<.001
Concomitant procedure				
Cerebrospinal fluid drainage	17 (3.3)	11 (2.8)	6 (4.5)	.3
Coronary artery bypass grafting	89 (17.1)	71 (18.3)	18 (13.6)	.2

Values are n (%) or median [quartile 1-quartile 3]. *BMI*, Body mass index; *LSCA*, left subclavian artery; *AV*, aortic valve; *ET*, elephant trunk; *CPB*, cardiopulmonary bypass; *HCA*, hypothermic circulatory arrest; *RCP*, retrograde cerebral perfusion; *ACP*, antegrade cerebral perfusion.

∗ Proximalization of repair was defined as the distal anastomosis performed at the anatomic level between the left common carotid and left subclavian arteries or more proximally.

### Early Outcomes

Patients with obesity did not have greater rates of composite adverse events (16.7% vs 17.7%, *P* = .8), operative death (12.9% vs 12.1%, *P* = .8), persistent stroke (3.8% vs 5.9%, *P* = .4), or any pulmonary complication (Table E2) than patients without obesity. However, they did have greater rates of transient spinal cord deficit (4.5% vs 1.3%, *P* = .02) and acute renal dysfunction (20.5% vs 11.3%, *P* = .008) and trended toward more frequent atrial arrythmia (39.4% vs 31.9%, *P* = .1) (Table 3). One patient without obesity developed persistent paraplegia after TAR repair with a frozen elephant trunk approach. Regarding the cause of operative death, there were few notable differences between patients with and without obesity (Table E3).

**Table 3:** Early outcomes stratified by obesity

Variable	All (N = 521)	BMI <30 (n = 389)	BMI ≥30 (n = 132)	*P*
Adverse event∗	91 (17.5)	69 (17.7)	22 (16.7)	.8
Operative death	64 (12.3)	47 (12.1)	17 (12.9)	.8
30-d death	47 (9.0)	34 (8.7)	13 (9.8)	.7
Neurologic deficit				
Stroke	35 (6.7)	29 (7.5)	6 (4.5)	.2
Persistent stroke†	28 (5.4)	23 (5.9)	5 (3.8)	.4
Persistent spinal cord deficit	7 (1.3)	5 (1.3)	2 (1.5)	.8
Transient spinal cord deficit‡	11 (2.1)	5 (1.3)	6 (4.5)	.02
Acute renal dysfunction	71 (13.6)	44 (11.3)	27 (20.5)	.008
Renal failure necessitating dialysis	42 (8.1)	32 (8.2)	10 (7.6)	.8
Persistent renal failure†	36 (6.9)	29 (7.5)	7 (5.3)	.4
Cardiac complication	262 (50.3)	195 (50.1)	67 (50.8)	.9
New-onset MI	6 (1.2)	3 (0.8)	3 (2.3)	.2
Arrythmia (any)	204 (39.2)	151 (38.8)	53 (40.2)	.8
Atrial arrythmia	176 (33.8)	124 (31.9)	52 (39.4)	.1
Pulmonary complication	249 (47.8)	192 (49.4)	57 (43.2)	.2
Respiratory failure	212 (40.7)	160 (41.1)	52 (39.4)	.7
Necessitating tracheostomy	78 (15.0)	59 (15.2)	19 (14.4)	.8
Bleeding requiring reoperation	17 (3.3)	16 (4.1)	1 (0.8)	.1
Sternal wound infection§	20 (3.8)	13 (3.3)	7 (5.3)	.4
Operative survivors	457 (87.7)	342 (87.9)	115 (87.1)	.8
Length of ICU stay, d	5 [3-12] (n = 440)	5 [3-12] (n = 327)	5 [2-12] (n = 113)	.6 –
Length of hospital stay, d	12 [9-22] (n = 449)	13 [9-22] (n = 334)	12 [8-23] (n = 115)	.5 –

Values are n (%) or median [quartile 1-quartile 3]. *BMI*, Body mass index; *MI*, myocardial infarction; *ICU*, intensive care unit.

∗ Defined as operative death or persistent (present at hospital discharge) stroke, paraplegia, paraparesis, or renal failure necessitating dialysis.

† Present at the time of hospital discharge or early death.

‡ A frozen elephant trunk approach was used in 8 of 11 patients (BMI ≥30; n = 5/6).

§ Sternal wounds were superficial (n = 19) or deep (n = 1; mediastinitis).

### Obesity by Class

There were few differences in characteristics of patients with obesity stratified by class. Notably, those with class 1 obesity (30 ≤ BMI < 35) were more likely to undergo reoperation and aortic root replacement than patients with class 2 (35 ≤ BMI < 40) and class 3 obesity (BMI ≥40) (Table 4).

**Table 4:** Comparative analysis of obesity stratified by class

Variable	Class 1 30 ≤ BMI < 35 (n = 94)	Class 2 35 ≤ BMI < 40 (n = 29)	Class 3 BMI ≥40 (n = 9)	Classes 2 and 3 BMI ≥35 (n = 38)	*P*∗
Age, y	65 [57-71]	64 [52-71]	59 [52-67]	64 [53-69]	.8
Male	68 (72)	18 (62)	7 (78)	25 (66)	.5
Heritable thoracic aortic disease	15 (16)	6 (21)	3 (33)	9 (24)	.3
Chronic proximal aortic dissection	51 (54)	13 (45)	2 (22)	15 (40)	.1
Diabetes	7 (7)	5 (17)	1 (11)	6 (16)	.1
Coronary artery disease	36 (38)	9 (31)	3 (33)	12 (32)	.5
Previous stroke	9 (10)	2 (7)	1 (11)	3 (8)	.8
BMI	32 [31-33]	36 [35-38]	43 [40-45]	37 [36-40]	<.001
Reoperation	57 (61)	10 (35)	5 (56)	15 (40)	.03
Femoral cannulation	9 (10)	3 (10)	0	3 (8)	.8
Aortic clamp time, min	54 [30-82]	53 [33-72]	51 [13-73]	52 [32-71]	.9
ACP time >30 min, n	74 (79)	21 (72)	9 (100)	30 (79)	>.9
Aortic root replacement	15 (16)	1 (3)	0	1 (3)	.03
Elephant trunk	70 (75)	23 (79)	5 (56)	28 (74)	.9
Frozen elephant trunk	21 (22)	10 (35)	3 (33)	13 (34)	.2
Adverse event (composite)	18 (19)	2 (7)	2 (22)	4 (11)	.2
Operative mortality	13 (14)	2 (7)	2 (22)	4 (11)	.6
Persistent stroke†	4 (4)	0	1 (11)	1 (3)	.7
Persistent paraparesis†	2 (2)	0	0	0	.4
Persistent paraplegia†	0	0	0	0	—
Persistent renal failure (dialysis)†	9 (10)	1 (3)	0	1 (3)	.2
Sternal wound infection	4 (4)	3 (10)	0	3 (8)	.4
Early survivors: ICU LOS, d	5 [2-12]	5 [3-11]	6 [2-13]	6 [2-12]	.6
Early survivors: overall LOS, d	11 [8-25]	15 [8-24]	12 [8-15]	14 [8-19]	.5

Values are n (%) or median [quartile 1-quartile 3]. Obesity is further grouped into class 1 (30 ≤ BMI < 35; n = 94 [71.2%]), class 2 (35 ≤ BMI < 40; n = 29 [21.9%]), and class 3 (BMI≥40; n = 9 [6.9%]). *BMI*, Body mass index; *ACP*, antegrade cerebral perfusion; *ICU*, intensive care unit; *LOS*, length of stay.

∗ *P* value compares class 1 versus a combined class 2 and 3.

† Present at the time of hospital discharge or early death.

## Findings From Modeling

**Figure E1:**
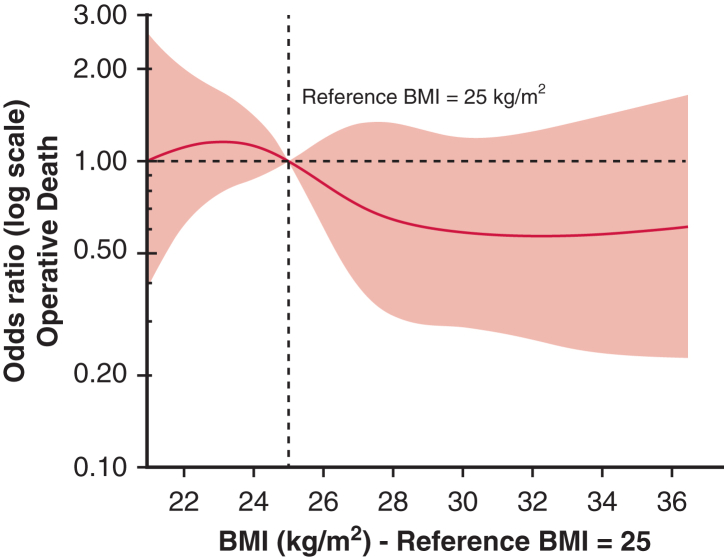
Restricted cubic spline from a multivariable logistic regression model depicting the predicted probability of operative death across the observed BMI range. The *horizontal dotted line* marks OR = 1.0, which represents no effect. Values above this line indicate greater risk, whereas values below indicate lower risk. The *vertical dotted line* marks BMI = 25 kg/m^2^ to indicate changes in predicted probability around this value. The predicted OR line is slightly above the no-effect line for BMI values <25 and below it for BMI values >25. The 95% CIs cross the no-effect line at all points, indicating no significant associations. *BMI*, Body mass index.

Overall, the multiple logistic regression model showed that certain preoperative and operative features, including chronic kidney disease (odds ratio [OR], 2.19, *P* = .01), pulmonary disease (OR, 2.50; *P* = .005), tobacco use (OR, 2.22; *P* = .03), and ACP time >30 minutes (OR, 2.69; *P* = .01), were better predictors of operative death than BMI ≥30 (OR, 0.99; *P* = .8) or age (OR, 1.01; *P* = .7) (Table 5). When BMI was modeled as a continuous variable to capture variations at lower BMI values, with 25 kg/m^2^ as the reference point, the analysis revealed no significant association between BMI and operative mortality. As Figure E1 shows, the OR remained close to 1 across the BMI range, and the confidence intervals consistently crossed the no-effect line, indicating no significant associations.

**Table 5:** Logistic regression model for predicting operative death in patients after elective total arch replacement

Variable	Odds ratio (95% CI)	*P* value
Age, y	1.01 (0.98-1.04)	.7
Body mass index ≥30	0.99 (0.93-1.06)	.8
Chronic kidney disease	2.19 (1.14-4.17)	.01
Pulmonary disease	2.50 (1.32-4.81)	.005
Current or former tobacco use	2.22 (1.07-4.97)	.03
Aortic clamp time, min	1.01 (1.00-1.01)	.058
ACP time >30 min, n	2.69 (1.22-6.62)	.01

*ACP*, Antegrade cerebral perfusion.

### Late Outcomes

**Figure 2:**
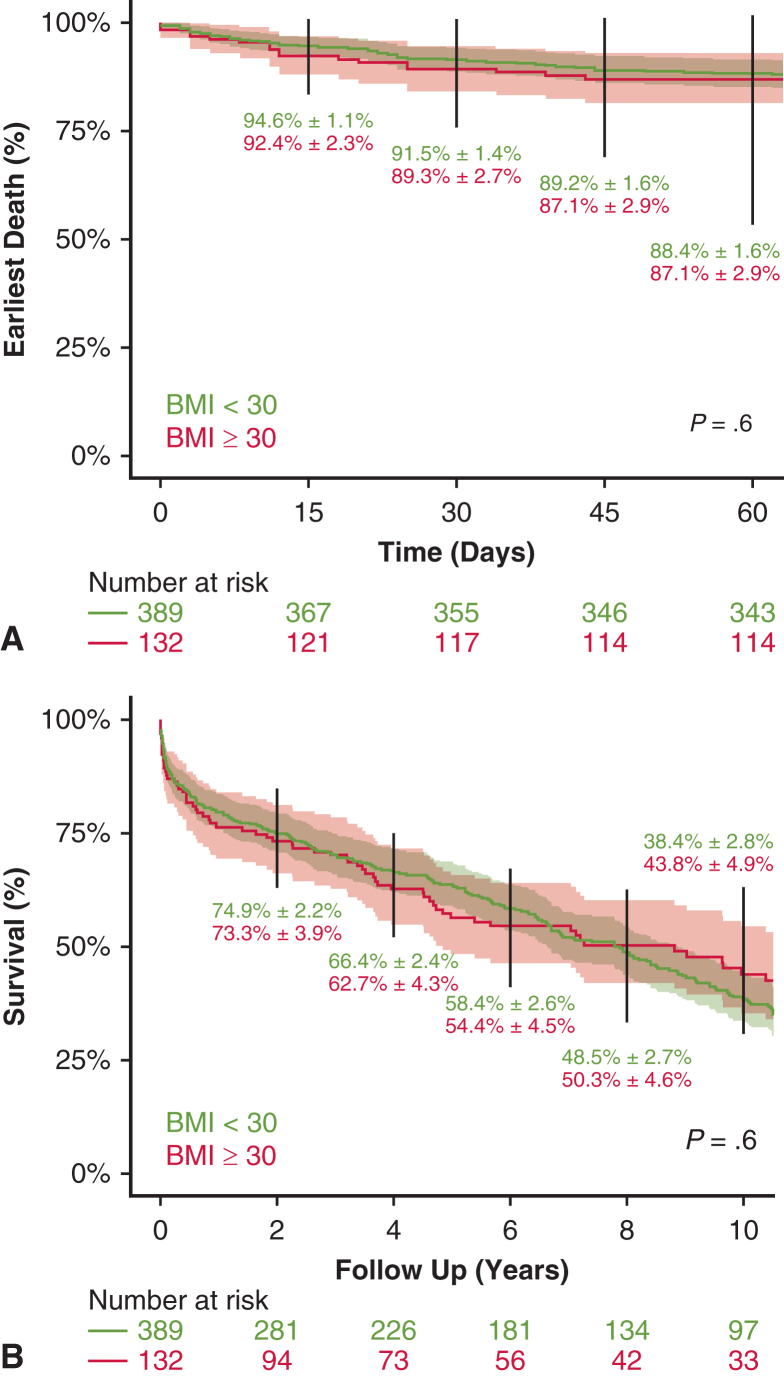
Kaplan-Meier estimated survival for all patients (n = 521) who underwent elective total aortic arch replacement, stratified by obesity (body mass index [BMI] ≥30). Shown are a snapshot of earliest death (≤60 days; A) and overall survival (≤10 years; B). The 95% CIs are shown as *shaded regions*.

Overall survival for patients with and without obesity was not significantly different (*P* = .6) (Figure 2).

## Discussion

We hypothesized that obesity would be associated with greater operative mortality and morbidity (eg, stroke) in patients who undergo elective TAR. Our results showed no substantial difference in perioperative mortality between patients with and without obesity, or among different degrees of obesity. Obstructive sleep apnea, a component of pulmonary disease, was more common in patients with obesity than without obesity. The durations of complex portions of the repair (eg, HCA and HCA with ACP) were longer in patients with obesity than in patients than without obesity. Patients with obesity had greater rates of transient spinal cord deficit and acute renal dysfunction but not of persistent spinal cord deficit or renal failure. It is possible that patients with obesity are more susceptible to spinal cord deficits after frozen elephant trunk approaches. Modeling showed that operative survival in patients with and without obesity was most closely related to pulmonary disease and chronic kidney disease.

This work is timely because the few studies that have investigated the relationship between obesity and aortic arch surgery dealt with nonelective cases (eg, aortic dissection repairs).^18^, ^19^, ^20^, ^21^ These studies associated obesity with postoperative severe hypoxemia,^18^ postoperative acute kidney injury,^21^ operative mortality, low cardiac output syndrome, pulmonary complications, and 5-year mortality.^19^ Although this study did not show worse permanent outcomes in patients with obesity, we nonetheless advocate optimizing preoperative variables such as obstructive sleep apnea ahead of surgery.

The absence of any significant association between obesity and mortality or persistent complications in patients who undergo TAR was surprising, because obesity is an important risk factor in nonelective TAR. This unexpected finding is important because it suggests that obesity should not preclude patients from undergoing elective TAR, even though surgeons can reasonably expect portions of these repairs to take longer in patients with obesity than in patients without obesity.

Limitations of this study include a heterogeneous cohort and highly variable repair strategies. Our study period was long because we elected to use a broad time frame to maximize cohort size. Fewer patients with obesity underwent repair before 2006, which may reflect a temporal shift in patient selection; additional bias could have arisen from referral patterns inherent to a tertiary care center. We had few patients with extreme obesity (class 3); these patients might not be referred for repair. In addition, we were unable to robustly evaluate surgical era as pertains to outcomes in patients with obesity. Our data collection shifted from retrospective to prospective in 2006. As a result, data on some outcomes—particularly operative mortality—were probably less complete in the earlier era, especially for patients who died after transfer to a long-term acute care facility. Likewise, others have suggested that surgical risk associated with TAR is underestimated when 30-day death is used as a primary measure because postoperative complications compromise survival in the early months of recovery.^22^

## Conclusions

TAR is a complex procedure with substantial operative mortality even when elective; nonetheless, the association between obesity and surgical risk in such patients, which was previously uncharacterized, appears to be minimal. Our results suggest that obesity alone should not disqualify patients from undergoing this operation.

## Conflict of Interest Statement

Dr Orozco-Sevilla participates in clinical trials for Gore Medical, Cook Medical, and Terumo Aortic and consults for Cook Medical. Dr Chatterjee has served on advisory boards for Edwards Lifesciences, Eagle Pharmaceuticals, La Jolla Pharmaceutical Company, and Baxter Lifesciences. Dr LeMaire serves as a consultant for Cerus. Dr Moon serves on an advisory board for Edwards Lifesciences. Dr Coselli consults for and participates in clinical trials for Terumo Aortic, Medtronic, Inc, and W.L. Gore & Associates and participates in clinical trials for Abbott Laboratories, Artivion, AstraZeneca, and Edwards Lifesciences. All other authors reported no conflicts of interest.

The *Journal* policy requires editors and reviewers to disclose conflicts of interest and to decline handling or reviewing manuscripts for which they may have a conflict of interest. The editors and reviewers of this article have no conflicts of interest.

## Figures and Tables

**Table E1:** Stratification of select variables by surgical era

Variables	All (N = 521)	Repair <2006 (n = 153)	Repair ≥2006 (n = 368)	*P*
Preoperative characteristics				
Age, y	65 [56-72]	66 [57-72]	65 [56-72]	.3
Heritable thoracic aortic disease	108 (20.7)	33 (21.6)	75 (20.4)	.8
Chronic proximal aortic dissection	238 (45.7)	55 (35.9)	183 (49.7)	.004
Proximal aortic diameter, max, cm	5.8 [5.1-6.6]	6.2 [5.5-7.0]	5.6 [5.0-6.3]	<.001
BMI <30	389 (74.7)	129 (84.3)	260 (70.7)	.001
BMI ≥30	132 (25.3)	24 (15.7)	108 (29.3)	.001
Diabetes	42 (8.1)	10 (6.5)	32 (8.7)	.4
Coronary artery disease	187 (35.9)	66 (43.1)	121 (32.9)	.03
Chronic kidney disease	136 (28.0)	46 (34.1)	90 (25.6)	.06
Pulmonary disease	200 (38.4)	55 (35.9)	145 (39.4)	.5
Obstructive sleep apnea	66 (12.7)	5 (3.3)	61 (16.6)	<.001
Current or former tobacco use	340 (65.3)	111 (72.5)	229 (62.2)	.02
Operative details				
Reoperation (redo incision)	270 (51.8)	60 (39.2)	210 (57.1)	<.001
Arterial cannulation site (initial)				
Direct aorta (ascending/arch)	86 (16.5)	71 (46.4)	15 (4.1)	<.001
Right axillary	229 (44.0)	15 (9.8)	214 (58.2)	<.001
Innominate	112 (21.5)	0	112 (30.4)	<.001
Right common carotid	20 (3.8)	0	20 (5.4)	.003
Femoral	68 (13.1)	64 (41.8)	4 (1.1)	<.001
Other or unknown	6 (1.2)	2 (1.3)	4 (1.1)	.8
Primary brachiocephalic reattachment				
Island (≥2 vessels)	278 (53.4)	134 (87.6)	144 (39.1)	<.001
Graft (≥2 vessels)	243 (46.6)	19 (12.4)	224 (60.9)	<.001
Other repair details				
Aortic clamp time, min	54 [27-80]	50 [25-71]	54 [28-87]	.9
Proximalization of repair (LCCA-LSCA)	221 (42.4)	31 (20.3)	190 (51.6)	<.001
ET	393 (75.4)	103 (67.3)	290 (78.8)	.006
Classic ET	266 (51.1)	103 (67.3)	163 (44.3)	<.001
Frozen ET∗	127 (24.4)	0	127 (34.5)	<.001
Lowest nasopharyngeal temp, °C	21 [16-23]	13 [11-15]	22 [20-24]	<.001
Cerebral perfusion				
None	35 (6.7)	34 (22.2)	1 (0.3)	<.001
Any RCP	105 (20.2)	101 (66.0)	4 (1.1)	<.001
Any ACP	400 (76.8)	33 (21.6)	367 (99.7)	<.001
ACP time >30 min, n	361 (69.3)	9 (5.9)	352 (95.7)	<.001
Early outcomes				
Adverse event†	91 (17.5)	19 (12.4)	72 (19.6)	.05
Operative death	64 (12.3)	11 (7.2)	53 (14.4)	.02
30-d death	47 (9.0)	10 (6.5)	37 (10.1)	.2
Persistent stroke‡	28 (5.4)	7 (4.6)	21 (5.7)	.6
Spinal cord deficit	15 (2.9)	2 (1.3)	13 (3.5)	.2
Transient spinal cord deficit	11 (2.1)	1 (0.7)	10 (2.7)	.1
Persistent paraplegia‡	1 (0.2)	0	1 (0.3)	.5
Acute renal dysfunction	71 (13.6)	9 (5.9)	62 (16.8)	<.001
Persistent renal failure‡	36 (6.9)	5 (3.3)	31 (8.4)	.04
Respiratory failure necessitating tracheostomy	78 (15.0)	22 (14.4)	56 (15.2)	.8
Arrythmia (any)	204 (39.2)	55 (35.9)	149 (40.5)	.3
Bleeding requiring reoperation	17 (3.3)	3 (2.0)	14 (3.8)	.3
Wound infection	21 (4.0)	9 (5.9)	12 (3.3)	.2
Sternal wound§	20 (3.8)	8 (5.2)	12 (3.3)	.3
Groin wound	2 (0.4)	1 (0.7)	1 (0.3)	.5

Values are n (%) or median [quartile 1-quartile 3]. *BMI*, Body mass index; *LCCA*, left common carotid artery; *LSCA*, left subclavian artery; *ET*, elephant trunk; *RCP*, retrograde cerebral perfusion; *ACP*, antegrade cerebral perfusion.

∗ Frozen elephant trunks include “homemade” approaches that rely on an elephant trunk graft with antegrade endograft deployment in addition to commercially available hybrid devices.

† Defined as operative death or persistent (present at hospital discharge) stroke, paraplegia, paraparesis, or renal failure necessitating dialysis.

‡ Present at the time of hospital discharge or early death; this patient did not have obesity.

§ Sternal wounds were superficial (n = 19) or deep (n = 1; mediastinitis).

**Table E2:** Expansion of pulmonary complications stratified by obesity

Variable	All (N = 521)	BMI <30 (n = 389)	BMI ≥30 (n = 132)	*P*
Pulmonary complication	249 (47.8)	192 (49.4)	57 (43.2)	.2
Respiratory failure	212 (40.7)	160 (41.1)	52 (39.4)	.7
Necessitating tracheostomy	78 (15.0)	59 (15.2)	19 (14.4)	.8
Need for reintubation	120 (23.0)	93 (23.9)	27 (20.5)	.4
Acute respiratory distress	28 (5.4)	21 (5.4)	7 (5.3)	>.9
Atelectasis necessitating bronchoscopy	47 (9.0)	34 (8.7)	13 (9.8)	.7
Chylothorax	0	0	0	–
Drainage of pleural effusion	1 (0.2)	0	1 (0.8)	.1
Pneumonia	43 (8.3)	1 (8.0)	12 (9.1)	.7
Evacuation of pneumothorax	15 (2.9)	13 (3.3)	2 (1.5)	.3
Pulmonary embolism	7 (1.3)	7 (1.8)	0	.1

Values are n (%). Pulmonary complication is a composite of all other variables in this table. In this set of patients, no patient developed postoperative chylothorax. Respiratory failure is a composite variable that describes ventilator dependence exceeding 48 hours before the time of discharge and includes failure necessitating tracheostomy and need for reintubation. *BMI*, Body mass index.

**Table E3:** Primary causes of operative death∗ for 64 patients

Variable	All (N = 64)	BMI <30 (n = 47)	BMI ≥30 (n = 17)
Multisystem organ failure	18	15	3
Multisystem organ failure with sepsis	5	3	2
Stroke with and without MSOF and other factors	10	7	3
Cardiac failure	9	4	5
Unknown†	7	7	0
Coagulopathy with and without MSOF and other factors	5	5	0
Pulmonary embolism	3	3	0
Cardiopulmonary failure, including sudden events	3	1	2
Respiratory failure	2	0	2
Myocardial infarction	2	1	1
Pneumonia	1	1	0
Rupture	1	1	0
Pericardial effusion after discharge to home	1	0	1
Heparin-induced thrombocytopenia	1	1	0
Fistula	1	1	0

Values are n. *BMI*, Body mass index; *MSOF*, multiple system organ failure.

∗ We defined operative death as death within 30 days of surgery or before final hospital discharge, including any death after transfer to another hospital.

† Unknown deaths typically occurred after transfer to a long-term acute care facility or after discharge home within 30 days of repair.
